# Multiple sclerosis and autoimmunity: a reappraisal of the evidence

**DOI:** 10.3389/fimmu.2026.1726369

**Published:** 2026-03-10

**Authors:** Marie Amigo, Monokesh K. Sen, James S. Dunn, David A. Mahns

**Affiliations:** 1 School of Medicine, Faculty of Health, Western Sydney University, Penrith, NSW, Australia; 2 Charles Perkins Centre, School of Medical Sciences, Faculty of Medicine and Health, The University of Sydney, Sydney, NSW, Australia

**Keywords:** autoimmune disease, autoimmunity, diagnostic criteria, multiple sclerosis, neurodegeneration, pathoetiology

## Abstract

Our understanding of the mechanisms underlying multiple sclerosis (MS) has advanced substantially over recent decades, yet the primary drivers of disease onset and progression remain unclear. Immune dysregulation, particularly antibody-mediated processes and lymphocyte activation, is widely recognised as central to MS pathogenesis, and immune-targeted therapies have improved the management of relapsing disease. However, neither self-antigens nor self-antibodies have been definitively identified. This leaves open a fundamental question: does immune activation initiate MS, or does it arise in response to earlier pathological events? Most of our current knowledge relies on extrapolating findings from artificially induced models, which are mechanistically informative but may be limited in explaining spontaneous onset and responses to neurodegeneration in MS. Furthermore, the recent reclassification of conditions such as MOGAD and NMOSD, previously considered within the MS spectrum, has prompted renewed reflection on longstanding assumptions regarding MS aetiology. In this review, we refine the definition of autoimmune disease (AD) and apply a systematic, criterion-based evaluation of MS, complemented by direct comparison with well-established autoimmune conditions. Unlike previous reviews, which have largely addressed this question in conceptual terms, this paper explicitly examines whether MS fulfils the defining features of autoimmunity. By doing so, we highlight conceptual and evidentiary gaps that remain unresolved. Clarifying whether MS should be defined as autoimmune is not merely semantic, but has important implications for experimental modelling, biomarker discovery, and therapeutic development. By encouraging exploration beyond the conventional autoimmune framework, this review seeks to support a more integrative understanding of disease mechanisms.

## Graphical Abstract

**Figure:**
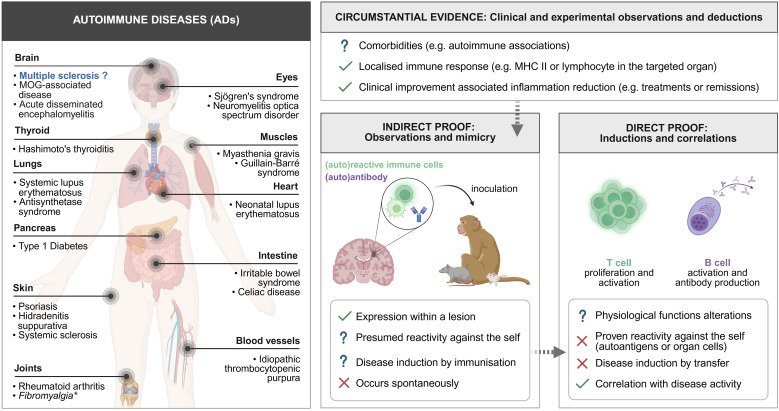

## 1 Multiple Sclerosis: a disease with an evolving definition

The historical journey of multiple sclerosis (MS) reflects a series of pivotal observations and scientific milestones. Early, ambiguous reports date back to 1395, when a 16-year-old girl developed symptoms consistent with the MS course (reviewed in (1)). It was in 1868 that Jean-Martin Charcot, the ‘Father of Neurology’, reported the first coherent story of MS pathology and introduced diagnostic criteria (2).

**Figure 1:**
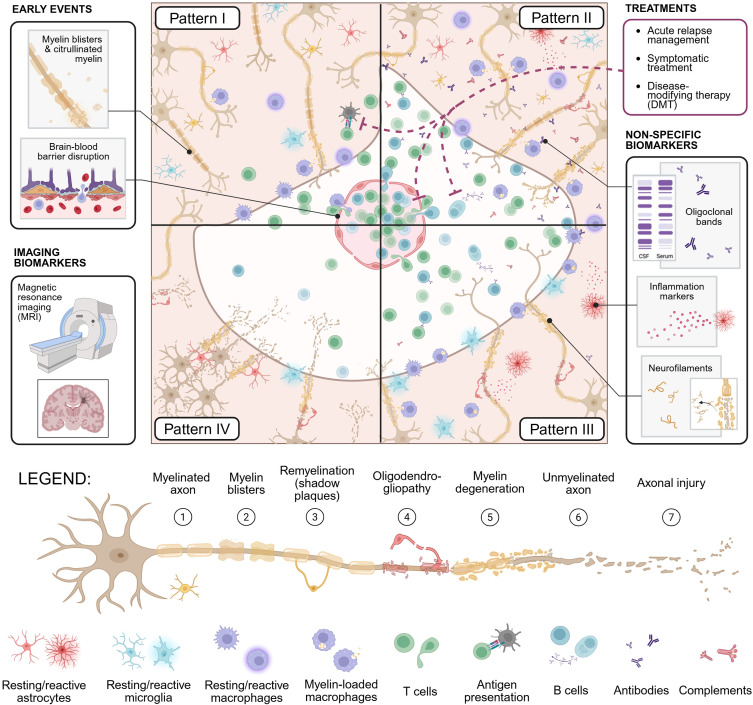
Schematic overview of the cellular and molecular markers associated with the four immunopathological patterns of MS lesions, including immune cells, Igs, complement deposition, and myelin debris (6). An MS lesion (white in the middle) can be cell-mediated, as in Pattern I, showing a small active lesion, localised around small veins and venules. A few immune cells (mostly T cells) infiltrate through a disrupted BBB (in red in the centre), and macrophages phagocytose myelin. Pattern II, the most prevalent pattern, is distinguished by prominent deposition of Igs and complement, together with increased myelin degradation products within macrophages; remyelinated shadow plaques are commonly observed in this pattern. Pattern III lesions are characterised by a less pronounced inflammatory response, with macrophages and activated microglia defining the border of the lesion, and features of distal oligodendrogliopathy (loss of myelin and apoptotic oligodendrocytes). Pattern IV shows a nearly complete loss of oligodendrocytes with an absence of remyelinated shadow plaques. Only a few inactive lymphocytes and macrophages remain in this lesion. The legend summarises the different immunological actors involved in the main stages observed in MS neurodegeneration. Early events and non-specific biomarkers have been at the core of MS research, contributing to the different aetiology hypotheses and autoimmune involvement in MS. Consequently, they also contributed to the model induction, diagnosis and treatments. Created in BioRender.

Characteristics of MS pathology include immune activation in the central nervous system (CNS) (3), blood-brain barrier (BBB) breach (4), and demyelination (5) (**Figure 1**). Yet, a fundamental question remains: does immune system dysregulation lead to an attack on the CNS, or does CNS damage initiate the immune response, exacerbating degeneration?

While demyelinating plaques consistently exhibit hallmarks of immune activity, including antibodies, lymphocytes and macrophages within CNS lesions, cerebrospinal fluid (CSF), blood, and lymphoid tissues (7), the presence of these immunological markers does not substantiate causality. Given its diverse symptomology and poorly understood mechanisms, MS diagnosis and classification have always been challenging and sometimes led to misdiagnosis (8). MS diagnosis relies on clinico-pathological grounds using magnetic resonance imaging (MRI) to prove disease dissemination in both time and space in the CNS (**Figure 1**) (9, 10).

Currently, there are two competing theories regarding the pathophysiology underlying the initiation of an autoimmune response against myelin and active lesion formation in MS (11–13).

- The ‘outside-in’ hypothesis is based on dysregulation and infiltration of autoreactive immune cells into the CNS through a disrupted BBB, leading to an inflammatory response against myelin and subsequent active lesion formation (14).
- In contrast, the ‘inside-out’ theory posits that MS is initiated by an underlying degeneration of oligodendrocytes, leading to demyelination and a subsequent autoimmune response within the CNS (15).

Despite compelling arguments on both sides, neither theory fully explains the triggering events of MS. Rodent models, particularly the experimental autoimmune encephalomyelitis mouse model (EAE) and cuprizone-fed mouse, have been instrumental in advancing our understanding of MS pathology, arguing for autoimmune involvement (16, 17).

Beyond T and B lymphocytes, animal models have suggested roles of other immune cell populations relevant to MS, including microglia, macrophages, dendritic cells, and innate lymphoid cells (18, 19). Animal models have also elucidated mechanisms of immune cell trafficking, antigen presentation, cytokine signaling, and interactions between the immune system and CNS-resident cells, many of which have directly informed hypotheses subsequently explored in human MS studies. These contributions have substantially advanced our understanding of inflammatory cascades and immune-mediated tissue damage in MS (20). Importantly, animal models have also prompted human studies to look beyond a purely inflammatory lens, supporting alternative scenarios such as early neurodegeneration and immune-neural interactions (21). However, the primary limitation of these models lies not in their capacity to model immune effector mechanisms but in their ability to faithfully recapitulate disease initiation and specificity in a spontaneous human disorder. Indeed, most preclinical models are inherently reductionist, designed around a limited set of disease features (e.g. T cell-driven CNS inflammation), and rely on artificial induction rather than spontaneous disease onset. Moreover, fundamental differences between human and rodent immune systems limit the translational value of such models. For example, humans express immunoglobulin (Ig) A receptor (FcαRI) and defensin-rich neutrophils, which are both absent in mice, and the proportion of circulating lymphocytes also differs (75-90% in mice, 30-50% in humans) (22). Redefining preclinical modelling is crucial to enhance translational treatment in humans.

As much of the current classification of MS as an autoimmune disease (AD) relies on such circumstantial and model-based evidence, critical investigations into the initiating factor(s) and their autoimmune nature are essential. To expand understanding of MS, continued evaluation of human-based evidence will be essential, alongside an openness to complementary frameworks that may extend beyond traditional autoimmune models. The heterogeneity of MS further complicates efforts to identify a unifying cause, as MS encompasses a spectrum of clinical and pathological phenotypes, suggesting multiple underlying mechanisms (23). The majority of patients initially present with relapsing–remitting MS (RRMS), which is characterised by presumed inflammatory activity, directing therapeutic development toward immune modulation (inflammation and immune reaction). However, if the inside-out model holds, these treatments may target subsequent symptoms rather than root causes. Even when treatments are designed to target processes thought to be central to disease pathology (**Figure 1**), the outcomes are partially curative, with disease progression still observed (24). It is also plausible that both paradigms contribute to MS pathogenesis, obscuring the distinction between onset processes and disease progression, and reinforcing a simplified autoimmune narrative without definitive causal proof.

## 2 Autoimmunity reconsidered: where does MS stand?

Before debating whether MS is autoimmune-driven or not, it is crucial to establish a well-defined foundational concept of autoimmunity. Several papers have extended Koch’s postulates “*an organism must be consistently found in a* sp*ecific form and arrangement within diseased tissue, and it should be capable of causing the disease when isolated and grown in pure culture”* (25) to a broader range of pathogenic agents, including self-proteins, to introduce autoimmunity (26–28). The addition of direct proofs, such as infection-induced transfer and recovery after inflammation reduction, leads to new criteria expanding the definition of ADs (29–31). This review gathers these criteria to provide an updated summary for ADs based on our interpretation. **Table 1** summarises those major criteria, supported by well-established ADs where the criterion has been validated, and the evidence supporting or conflicting with these criteria in MS onset and progression. This table aims to provide a template to confirm the autoimmune nature of a disease.

**Table 1:** Criteria for autoimmune classification: evidence in well-established autoimmune diseases (ADs) and MS.

Autoimmune disease (AD) criteria		ADs examples	MS	
			Supportive	Conflicting/inconclusive
Circumstantial evidence	Other autoimmune associations	Sjögren’s syndrome and RA (32)	(33, 34)	(35)
	Altered expression of MHC II antigens in the targeted organ	T1D (36)	(37)	(38)
	Lymphocytic infiltration in the targeted organ	RA (39), SLE/NMOSF (40)	(41)	(42)
	Good response to immune suppression	MG, NMOSD (43)	(44, 45)	(46, 47)
	Inflammation reduction associated with clinical improvement	RA (48)	(49–51)	None
Indirect proof	Disease reproduction by experimental immunisation	MG (52)	(53, 54)	(55, 56)
	Disease reproduction by idiotypes	Celiac disease (57)	(58)	(19, 55)
	Spontaneous animal models (genetically induced)	T1D (59) ADEM (60)	(61, 62)	(61, 63)
	Antibodies localised in a lesion	Hashimoto’s Thyroiditis (64)	(6, 65)	(6, 66)
	Activated immune cells in a lesion	Hashimoto’s Thyroiditis (67)	(7, 68)	(6, 69)
	Autoreactive T cells	T1D (70)	(71, 72)	(66)
	Autoantibodies	RA (73) MOGAD (74)	(75, 76)	(77, 78)
Direct proof	Circulating antibodies which alter a physiological function	NMOSD (79)	(80)	(81, 82)
	Disease reproduction by antibody transfer: 1. Experimental transfer 2. Maternal transfer 3. Animal transfer	1. Idiopathic thrombocytopenia (83) 2. MG (84) 3. NMOSD (85)	1. None 2. None 3 (86, 87).	1. None 2 (88). 3 (19, 55).
	Direct correlation between autoantibodies and disease activity	Hidradenitis Suppurativa (89)	(90, 91)	(92)
	Direct correlation between T cells and disease activity	SLE (93)	(94)	(95)
	*In vitro* T cell proliferation in response to autoantigen	T1D (96)	(72)	(97, 98)
	*In vitro* T cell cytotoxicity against target organ cells	Guillain-Barré syndrome (99)	(100)	(101)
	*In vitro* T cell transference to immunodeficient mice	Systemic sclerosis (102)	(103, 104)	(55, 104)

An immunological disease can be classified as autoimmune-triggered and -driven if it meets several criteria. Derived from Koch’s postulates (25) and extended to a broader range of pathogens (29–31), this table suggests an elaborated definition for autoimmune diseases (ADs). These criteria are categorised based on the strength of the correlation between the pathology onset and autoimmune mechanisms. They can either deduce/presume an association between inflammation and disease progression (circumstantial evidence), indicate an indirect link/relation with autoimmunity in a diseased model (indirect proofs), or provide direct evidence of the disease causation by an autoimmune response (direct proofs). This table illustrates the different criteria by giving AD examples (MG, myasthenia gravis; RA, rheumatoid arthritis; T1D, type 1 diabetes; SLE, systemic lupus erythematosus; NMOSD, neuromyelitis optica spectrum disease; MOGAD, myelin oligodendrocyte glycoprotein-associated disease; ADEM, acute disseminated encephalomyelitis). Additionally, it reviews supportive and inconclusive elements of each criterion in MS.

Whilst the definition of ADs has been debatable, it is generally characterised by the loss of immune tolerance, leading to tissue damage in the absence of any other evident cause (31, 105). More than 100 ADs have been identified (106) and more will come once a clear definition is widely integrated.

We suggest that broad or simplified application of the autoimmunity definition, without explicit consideration of different levels of evidence (circumstantial, indirect and direct), may influence the investigation of several diseases, including MS, potentially narrowing the scope of alternative explanatory models (107). Indeed, diseases such as fibromyalgia have *sometimes* been considered as autoimmune, while several criteria required to fulfil the definition of autoimmunity have not been met yet (108). Additionally, other established ADs, such as systemic lupus erythematosus (SLE), do not fully meet all these criteria (e.g. no single defining target) (109), underscoring the incomplete or conflicting evidence observed across several diseases. Thus, a clear and shared definition adopted by the research community is essential to determine whether MS can be considered an AD in its initiation and progression.

Several reviews have previously questioned the autoimmune paradigm in MS (107, 110–112); however, these discussions have most often remained descriptive or conceptual, without systematically evaluating MS against the defining criteria of AD. In this context, the present review offers a structured and complementary perspective on ADs and, more specifically, on the onset of MS. Although numerous studies have examined these criteria in MS (**Table 1**), the available evidence is frequently mixed or incomplete, often reflecting limited cohort sizes or the need for further experimental validation. By bringing these findings together within a unified, criterion-based framework, this review aims to clarify the extent to which MS aligns with established autoimmune paradigms and to highlight areas where further investigation is most likely to be informative.

Against this background, whether MS is defined as an AD or not is merely a matter of terminology. Disease classification directly influences the development of experimental models, the prioritisation of biomarkers, and the design and interpretation of therapeutic strategies. An autoimmune-first framework naturally emphasises immune triggers, antigen specificity, and inflammatory mechanisms, whereas alternative frameworks may prompt greater investigation of early neurodegenerative, metabolic, or homeostatic disturbances that precede immune activation.

### 2.1 Circumstantial evidence

Unlike direct or indirect proof, circumstantial evidence comes from presumed autoimmune involvement and clinical observations. It does not directly show an immune attack at disease onset; rather, it suggests a possible link with the disease progression. While circumstantial evidence alone isn’t enough to classify a disease as autoimmune, it is a crucial first step in informing and guiding further investigation.

#### 2.1.1 Other autoimmune associations

Comorbidity with another autoimmune condition has been used as evidence to present an impaired immune tolerance (113). Two diseases can co-occur either by chance or by shared disease processes, where the presence of one disease is responsible for another, suggesting a potential overlap of autoimmune-driven pathomechanisms. Several diseases with autoimmune features seem to have symptom comorbidity, such as rheumatoid arthritis (RA) and Sjögren’s syndrome (32).

In the context of MS, several reviews have highlighted the coexistence of multiple disorders, including autoimmune/inflammatory pathologies, suggesting common genetic and environmental risk factors (114). Indeed, there have been occasional case reports demonstrating MS association with psoriasis, thyroid disease (33) or inflammatory bowel disease (34). Contrastingly, population-based data show that women with MS are no more likely to have specific autoimmune comorbidities than women without MS (35). Comorbidity studies need to be considered with extra care, considering the cohort diversity or the publication bias leading to potential overestimation of disease prevalence.

Therefore, this circumstantial evidence can only be validated if considered carefully and receives further investigation.

#### 2.1.2 Aberrant expression of MHC II antigens or lymphocytic infiltration in the targeted organ

Changes in major histocompatibility complex 2 (MHC II) expression or lymphocyte infiltration within a targeted organ support a localised immune response against self-proteins (115). A strong statistical association exists between certain MHC haplotypes and ADs such as Type 1 Diabetes (T1D), where aberrant MHC II expression on affected tissues plays a crucial role (36).

Among the MS-associated genes (developed in section 2.2.2), MHC II genes, particularly the HLA-DRB1*15:01 allele, have the strongest gene association with MS (7, 116, 117). However, despite its presence in multiple ADs (118, 119), the precise mechanisms by which human leukocyte antigen (HLA) molecules are involved in disease pathogenesis remain unclear. This complexity is largely due to the extreme polymorphism within HLA genes and the presence of multiple independent risk alleles across diverse human populations (38). Notably, MHC II proteins are strongly upregulated on activated microglia and macrophages within MS lesions, reinforcing their involvement in disease pathology (37). However, inter-individual and population-level variability in HLA haplotype expression introduces heterogeneity that may partly explain inconsistencies in functional studies (120).

In parallel, lymphocytic infiltration in the targeted organ indirectly reflects their potential organ-selective self-reactivity, as observed in the synovial fluid and tonsils of people with RA (39). Additionally, brain-infiltrated antibodies contribute to brain pathologies such as SLE and neuromyelitis optica spectrum disease (NMOSD), highlighting potential overlapping/conserved mechanisms across neurological autoimmune disorders (40).

In MS, it is well recognised that B and T cells, particularly CD8^+^ and CD4^+^ T cells, infiltrate the CNS through a disrupted BBB and react against the brain (**Figure 1**) (41). However, the precise epitopes these T cells recognise remain unidentified. Activated lymphocytes are present in MS lesions and play a crucial role in the immune reaction against proteins expressed in the CNS (121). In contrast, purely cortical lesions have been reported to lack prominent lymphocytic and macrophagic inflammatory infiltrates, contradicting their role in lesion formation (42).

Thus, while there is substantial evidence of self-recognition mechanisms in MS brain lesions, it remains uncertain whether these mechanisms originate from a primary autoimmune process or are secondary to another initiating event.

#### 2.1.3 Good response to immune suppression

Promoting pharmacological immunosuppression in ADs is a standard strategy in disease management. Therefore, a good response (e.g. clinical improvement) to immune suppression is suggestive of circumstantial autoimmunity. Immunosuppression approaches can be used to treat neurological disorders such as myasthenia gravis (MG) and NMOSD (43).

In MS, management strategies primarily focus on the treatment of acute relapses, symptomatic management, and reduction of disease activity through disease-modifying therapies (DMTs, **Figure 1**) (122–124):

- Acute relapse management prioritises the identification and treatment of concomitant conditions that may mimic or exacerbate relapse symptoms (e.g. urinary tract infections) (125).
- The symptomatic treatments act on significant and disabling symptoms by targeting contributing or co-existing factors, such as pain or anaemia (126).
- DMTs modulate or suppress the immune function to reduce inflammatory disease activity and relapse frequency (127).

While DMTs effectively reduce relapse rates and CNS inflammation, their impact on the neurodegenerative/non-inflammatory aspects of MS remains limited (46, 128). Current MS therapies predominantly target non-specifically adaptive immune components, such as T and B cells, to modulate the inflammatory disease activity (relapse rate, CNS lesion, disability accumulation). For instance, natalizumab (*Tysabri*) inhibits lymphocyte migration into the CNS and is highly effective in reducing relapse rate and relapse-associated disability accrual in RRMS (44). However, its clinical use is constrained by the risk of progressive multifocal leukoencephalopathy, necessitating careful patient monitoring and personalised treatment plans (47). Similarly, B cell-depleting monoclonal antibodies such as ocrelizumab (*Ocrevus*) have demonstrated clinically meaningful efficacy in both RRMS and in primary progressive MS (PPMS), supporting the role for B cells in early and inflammatory disease mechanisms (45). Additionally, these therapies have demonstrated great efficiency with a substantial reduction of MRI lesions up to 90% (129, 130).

In contrast, while most immunosuppressive approaches reduce relapse rate in RRMS, they show limited efficacy in slowing progression independent of relapse activity (PIRA) (46, 122). Consistently, these treatments are more effective in relapsing than progressive forms of MS, indicating that additional or distinct mechanisms likely contribute to non-inflammatory progression (131). Importantly, the historical misclassification of distinct demyelinating diseases (e.g. NMOSD or myelin oligodendrocyte glycoprotein antibody-associated disease [MOGAD]) as MS may have confounded earlier research and therapeutic studies, potentially inflating or obscuring treatment effects attributed to MS pathology. Finally, the limited translational efficacy between animal models and human studies further complicates treatment investigations (19, 132).

Therefore, although promising treatments have shown clinically meaningful long-term benefits (133), reducing relapse-associated disability accrual by approximately 20-70%, depending on the agent, their variable efficacy underscores the need to better understand mechanisms driving non-inflammatory progression (e.g. PIRA) and inter-individual treatment response.

#### 2.1.4 Reduction of inflammation associated with clinical improvement

The correlation between inflammation reduction and clinical improvement suggests a central immune involvement in pathology. Some fluctuating ADs characterised by flare-ups and remission are a good example to study the correlation between inflammation and disease activity. For instance, during RA remission, there is an increase in macrophage subpopulations expressing unique transcriptomic signatures enriched in negative regulators of inflammation (48).

Crucially, while immune cell activation is frequently reported as a driver of MS pathology, the molecular mechanisms leading to disease fluctuation remain insufficiently explored (134). There is a T and B cell balance shift, from pro-inflammatory to regulatory, inducing a reduction of inflammation overall during the remission phase (49, 50). Regulatory T and B cells (Tregs and Bregs) limit the immune response by producing immunosuppressive cytokines, cell contact-dependent cytolysis, and metabolic disruption (135). A decrease in Tregs and Bregs is believed to facilitate MS development and/or inflammatory activity, and to be correlated with disease severity (51, 136).

Additionally, some strategies aim to reduce, without completely suppressing the immune function by supporting the body’s natural healing, whether by stimulating the microbiome (diet, transplantations or pre- and pro-biotics (137)), improving quality of life and self-care (sleep, stress or physical activity (138)) or restoring homeostasis with supplementation (vitamins or anti-inflammatory components (139, 140)). Corticosteroid therapy is also commonly used to treat relapses in people with RRMS. It reduces activated dendritic and T cells, while increasing Tregs, thereby inducing overall inflammation reduction associated with clinical improvement (141). Understanding the molecular foundations of both the attack and the recovery may provide new insight into the role of the immune system in MS.

Importantly, it is evident that MS pathology is closely linked to inflammation, and a reduction of the latter appears to result in some clinical improvement in RRMS (mostly based on relapse frequency, disability score and brain volume loss), validating this criterion. However, further studies are needed to identify the factors and mechanisms associated with this improvement in RRMS and extend it to progressive forms, which will enhance our understanding of the disease and promote remission.

### 2.2 Indirect proofs

Indirect proofs differ from others by focusing on molecular evidence and its transferability to animals, rather than direct causality with the disease. A significant new approach to establishing the autoimmune origin of a specific human disease involves isolating autoantibodies or self-reactive T cells from the organs that are primarily affected by the autoimmune condition. This method contributes to building a chain of evidence to support the link between autoimmunity and disease.

#### 2.2.1 Disease reproduction by experimental immunisation or by idiotypes

Experimental immunisation using human analogous autoantigens in an animal model would induce antibody or T cell reactions, producing characteristic lesions, followed by the occurrence of similar features observed in the associated disease. For instance, individuals with MG express autoantibodies that recognise acetylcholine receptor (AchR) at the neuromuscular junction, inducing muscle weakness and fatigue (142). The injection of the AchR analogue in an animal model can induce the production of anti-AchR autoantibodies and a phenotype similar to MG (52).

Multiple antigens can mimic the MS process in animal models with relative uniformity, leading to ambiguity in identifying the most relevant antigen for disease onset in humans. Indeed, as noted in section 2.1.3, certain antibody responses are now considered to underpin distinct disease processes (e.g. MOGAD). Due to the lack of specific autoantigens associated with MS, fully validating this criterion has been impossible (55, 56). However, if myelin proteins (myelin basic protein [MBP], myelin oligodendrocyte glycoprotein [MOG], proteolipid protein [PLP]) are the targeted autoantigens in MS, EAE models would be a good example for debating this criterion. EAE can be induced through various protocols involving multiple injections of purified protein, adjuvants, and BBB toxins (53, 54). Lastly, EAE clinical and pathological features vary widely depending on the protocol and animal used (species, strain, gender, age…), highlighting the model’s complexity and limitations (20).

Another way to reproduce a disease is to inject full antibodies or idiotype-specific antibodies into animals, leading to symptoms mirroring those seen in human ADs. An idiotype is a unique set of molecular features found in the variable region of an antibody (or T cell receptor [TCR]) that is specific to that antibody’s unique antigen-binding site (143). This fingerprint allows each antibody or receptor to recognise a unique part of a pathogenic substance (144). In the context of some ADs, such as Celiac disease, idiotypic manipulation can induce anti-idiotypic responses and symptom development in naive mice (57).

In MS, it has been suggested that the initial triggering event is an infection or a series of infections outside the CNS that induce the generation of B cells carrying rare immunogenic idiotypes and that these B cells later engage in idiotype-driven T-B cell collaboration in the CNS (145, 146). The injection of idiotypes against MBP found in the CSF of people with MS induces encephalomyelitis in mice (58). However, despite the promising development of anti-idiotype and its effect on T cell-mediated tissue damage, this alternative EAE model doesn’t reflect the full complexity of the disease (55) and the etiological role of myelin proteins in MS has not yet been established.

While the methods and techniques have been robustly developed, the absence of a clearly defined MS-specific antigen limits definitive interpretation. Consequently, without an identified MS-specific antibody, these results, while showing promise, remain supportive but not conclusive.

#### 2.2.2 Spontaneous animal models (e.g. genetically modified)

In the case of certain ADs, a spontaneous animal model can be genetically induced to mimic the pathology (147). Indeed, mutations on genes encoding for antigen recognition (MHC molecules, proteins involved with antigen processing, and TCRs) or humoral response (proteins inducing Igs and cytokine production) will induce autoimmunity (105). The Non-Obese Diabetic (NOD) mouse is a spontaneous model resulting from a combination of genetic mutations affecting T cell regulation, making them prone to autoimmunity, leading to a T1D-like phenotype (59).

In MS, although no equivalent spontaneous genetic model exists, human genetic studies provide strong evidence for immune involvement in disease susceptibility. Beyond HLA associations, large-scale genome-wide association studies (GWAS) have identified more than 200 risk variants for MS (148–151), the majority of which map to genes involved in immune-related pathways, including antigen presentation, lymphocyte activation, cytokine signaling, and innate immune pathways (152, 153). Notably, some of these variants/pathways are shared with other ADs (154, 155), supporting overlapping genetic architectures across immune-mediated diseases. In parallel, large transcriptomic analyses of untreated people with MS have revealed persistent immune gene expression signatures despite the absence of overt inflammatory disease activity (156). While this genetic enrichment provides strong evidence for immune involvement in MS susceptibility, it does not, in itself, establish immune dysregulation as the initiating event of the disease.

To explore causal mechanisms, transgenic mice affecting T or B cells by deletion or over-expression of relevant genes (e.g. TCRs, MHC, cytokines, neurotrophic factors and/or receptors) have *sometimes* provided spontaneous models of CNS inflammation with diverse incidence, phenotypical and pathological patterns (61, 157). These models have contributed substantially to our understanding of immune activation and effector mechanisms relevant to MS. However, they are constrained by their experimental design and by the absence of a clearly defined disease-initiating trigger.

As discussed earlier, MS is a human disease difficult to mimic without knowing the initiating event. While there is no actual spontaneous model of MS in nature, Japanese macaque encephalomyelitis (JME) is a spontaneous and naturally occurring, demyelinating disease of the non-human primate displaying features comparable with MS (demyelination [MRI, focal lesions], immune mechanisms [CD4^+^ and CD8^+^ brain-infiltration and oligoclonal bands in CSF] and motor symptoms [paralysis, ataxia and ocular motor paresis]) (62, 158). Whilst JME seems to be an attractive model to study MS onset, the abnormalities resemble acute disseminated encephalomyelitis (ADEM) more than MS (60, 63). Further work is required to study the longitudinal associations between JME and MS.

Therefore, although encouraging work has been conducted leading towards autoimmune patterns, additional research is required to identify MS-specific genes and develop a spontaneous model to validate this criterion.

#### 2.2.3 Antibodies or activated immune cells localised in a lesion

The presence of key immune factors (e.g. antibodies and activated immune cells) in a lesion strongly suggests, indirectly, that the immune system is reacting selectively against a protein expressed in that lesion. For example, Hashimoto’s thyroiditis is characterised by antibodies against thyroid peroxidase and thyroglobulin (64). Additionally, lymphocytic infiltration, primarily consisting of T and B cells, occurs within the thyroid gland, leading to the gradual destruction of thyroid tissue, which demonstrates the organ specificity of the immune response (67).

Circulating and lesion-localised antibodies have been found in approximately 60% of individuals with MS and were previously used as a diagnostic criterion (e.g. oligoclonal bands, **Figure 1**) (65, 159). While these antibodies are the hallmark of the immunopathological Pattern II of MS lesions (**Figure 1**) (6), their non-selective and non-specific presence in MS has led to their removal from diagnostic criteria (66, 160). Additionally, although there have been several proposed candidate antigens (reviewed by (161, 162)), none have been confirmed yet, leaving unclear the precise mechanisms that induce immune activation in the periphery, its migration, and its response in the CNS in MS.

Studies have identified activated immune cells (e.g. lymphocytes, macrophages and microglia – **Figure 1**) in MS brain lesions (7). In contrast, certain lesion types, such as early-stage and chronic active lesions, exhibit demyelination with minimal lymphocytic infiltration (6), as exemplified by pattern IV in **Figure 1**. Instead, these lesions show prominent microglial activation and macrophage-mediated debris clearance, suggesting a prominent role for innate immunity (68, 69). Additionally, their presence in early lesions before any lymphocytic infiltration supports their involvement as primary responders to myelin injury rather than secondary participants in an adaptive immune attack (110, 163).

Regardless of whether these antibodies or activated immune cells contribute to disease onset, their presence in MS lesions is well established, supporting this criterion. Therefore, based on these findings, it can be inferred that the immune system is reacting against the self, yet further investigation is required to clarify the causal role in the onset, as the lesions generally arise once the disease process is already underway.

#### 2.2.4 Autoreactive T cells or autoantibodies

The presence of autoreactive T cells or autoantibodies, hallmarks of autoimmunity, serves as an important indicator of autoimmune mechanisms. Autoreactive T cells play a crucial and unique role in each AD (164), recognising a selective type of autoantigens, such as islet autoantigens in T1D (70).

In MS, several studies have reported a higher frequency of autoreactive T cell precursors recognising myelin antigens such as MBP and PLP, compared to healthy individuals (71, 72). However, similar immune reactivity has been observed in healthy controls (66) and attempts to isolate T cell clones from the brains of people with MS failed to show reactivity against myelin proteins. In contrast, both circulating and lesional CD8^+^ T cells express unique TCR specific to an as-yet-undefined antigen, suggesting their immunologic response is brain-selective (165) and can be detected for up to five years after recognition (41). This implies ongoing exposure to a persistent antigen and/or the development of strong and enduring T cell memory specific to that antigen (**Figure 1**).

In parallel, autoantibodies can be detected years before clinical diagnosis in AD, such as T1D (anti-insulinoma-associated antigen-2 antibodies) and RA (anti-citrullinated protein antibodies) (73, 166). However, while these autoantibodies can precede disease onset, they do not consistently predict whether an individual will develop the corresponding AD, making them an indirect marker of underlying damage and disease risk rather than definitive proof of causality (167, 168). For instance, some at-risk individuals exhibit predictive biomarkers for RA yet never develop inflammatory arthritis (169).

Previously, elevated serum antibody levels and physiological changes suggested that anti-MOG was a specific autoantibody for MS (170) and that levels of anti-MOG expression could serve as a prognostic biomarker in paediatric MS (171). Consequently, it was believed to validate this criterion in MS. Now, MOG is exclusively associated with MOGAD (74, 77). While these recent updates invalidate this criterion for MS, they should raise questions about the actual findings relying on MOG as the main MS actor. The intrathecal production of antibodies and the presence of oligoclonal bands in the CSF of people with MS, combined with reactive B cell infiltration in the CNS, suggest their participation in the development of the disease (172, 173) and their specificity to brain antigens such as myelin proteins (75, 80). However, due to their broad reactivity, the Igs present in the CSF of people with MS may consist of “nonsense” antibodies that do not contribute to the development of MS (78, 174). While various studies have sought to identify MS-associated autoantibodies (76, 175), none have been validated as diagnostic markers. Many proposed autoantigens originate from EAE models, which have heavily biased MS research toward myelin proteins and CD4^+^ T cell responses despite the absence of definitive evidence linking these antigens to disease onset in humans.

Lastly, these findings do not definitively prove that the antigen has an endogenous (self-protein) or exogenous (virus, bacteria) origin. Indeed, while MS-associated MHC II molecules exhibit specificity toward myelin proteins (71, 176), this does not preclude the possibility that the initial immune response was triggered by viral antigen presentation, subsequently cross-reacting with a self-protein, leading to the generation of autoantibodies (177, 178). This concept of autoimmunity, known as molecular mimicry, has long been proposed as a contributing factor in MS pathology, with potential cross reactions between human papillomavirus 6 (HPV-6) and MBP, as well as Epstein-Barr virus (EBV) and glial cell adhesion molecule (Glial CAM) (179–181). Epidemiological studies have consistently reported a strong association between EBV infection and MS, with nearly all individuals who develop MS showing evidence of prior EBV seroconversion, often preceding clinical disease onset (182). While these findings support EBV as a major environmental risk factor in MS, its relationship with disease course remains less clear and, in some studies, controversial (183). Importantly, EBV has also been implicated as a risk factor for other ADs, including SLE, by reprogramming B cells into autoreactive B cells driving autoimmunity (184, 185). Taken together, these observations suggest that molecular mimicry, potentially via EBV, could participate in the initiation of ADs, including MS. Further research is warranted to explore these connections and the actual involvement in the disease onset.

Crucially, autoimmune mechanisms in MS likely contribute to disease development, but their presence does not necessarily establish autoimmunity as the primary trigger. While MS shares several features with classical autoimmune diseases, it does not unequivocally meet all established criteria. A deeper exploration of immune system involvement through direct proof is essential to refine our understanding of MS aetiology further.

### 2.3 Direct proofs

This section focuses on criteria highlighting the causality between the autoimmune response and disease initiation. In contrast with the previous criteria, direct proof aims to show that an immune alteration is responsible for the clinical observations, mostly relying on disease induction and subsequent correlations.

#### 2.3.1 Circulating antibodies which alter a physiological function

A primary criterion of an AD is that a precise antigen is present in all patients and alters (stimulates or reduces) a specific biological function, subsequently inducing the pathology. For instance, NMOSD is characterised by the expression of anti-aquaporin 4 antibodies (AQP4), which induce AQP4 internalisation and alter its expression at the interface between astrocyte end-feet and endothelial cells, resulting in BBB disruption and neuroinflammation (79).

Many associations between MS and antibodies have been reported, frequently targeting myelin proteins and their functions. It is largely undisputed that there are changes in the isoform composition and structure of myelin proteins in MS (80). However, pathological analyses of MS lesions suggest that early myelin abnormalities begin at the inner lamellae rather than the outer layers, which are more readily accessible to immune attack (186). Furthermore, recent studies highlight early pathological changes in normal-appearing white matter of people with MS, including increased myelin blistering, post-translational myelin modifications, and microglial responses against citrullinated MBP (81, 82). These findings suggest that MS pathology may begin long before overt immune involvement, challenging the traditional autoimmune model.

The presence of multiple proposed antigens without any single antigen proving to be MS-specific, together with evidence of early myelin abnormalities, underscores the uncertainty surrounding MS onset and suggests that this primary criterion for classification as an autoimmune disease remains incompletely satisfied.

#### 2.3.2 Disease reproduction by antibody transfer

Immune responses and disease replication can occur through plasma exchange or passive transfer. This transfer can be experimental (human to human), maternal (human or animal), or animal (here focusing on human to animal).

One of the rare *experimental transfer*s involved nonthrombocytopenic volunteers injected with whole blood or plasma from patients with idiopathic thrombocytopenia. These individuals reproduced the disease, characterised by a prompt decrease in circulating platelets and increased bleeding tendency (bruising or petechiae), lasting for up to seven days (83). For obvious reasons and risks, this approach has never been reported using plasma from people with MS.

In MG, *maternal transfer* results from placental transmission of autoantibodies (anti-AchR) from an afflicted mother to the foetus, and causes transitory muscle weakness in the neonate (84). So far, there is no evidence of a maternal antibody transfer in MS, but it has been shown that maternal antibodies could be against placental proteins (88). Parallel studies in rats confirmed that antibodies against neurofascin (a cell adhesion molecule located at the nodes of Ranvier) can cross the placenta and bind to the developing cortex and cerebellum. However, this transfer does not appear to have adverse effects on the offspring (187).

As an example of *animal transfer*, the injection of AQP4 antibodies from humans into animals *sometimes* triggers brain lesions similar to those in NMOSD (85). It has been reported that the passive transference of plasma from humans to animal models could trigger and exacerbate some symptoms similar to MS. Compared with Igs derived from healthy controls, MS-derived Igs have been reported to increase disease activity and inflammatory infiltrates in the brain and CSF in EAE (86, 87). This suggests that people with MS may express Igs targeting brain proteins, which could be linked to disease severity. However, in these studies, antibody-mediated exacerbation occurred in the context of pre-established EAE, following active disease induction. As a result, it remains difficult to determine whether MS Igs directly drive the observed effects or whether they act to amplify an ongoing, experimentally induced immune process. Additionally, the EAE model is often induced by peripheral inoculation with myelin-related components, which are not specific to MS (e.g. MOGAD), and inflammation dominates restricted regions depending on the used antigen (e.g. MBP in lumbar regions, MOG in brainstem) (55).

It is therefore likely that the identification of a definitive MS-specific antigen would substantially clarify the role of humoral immunity in MS and help reconcile findings from experimental models with human disease. Accordingly, in the absence of a confirmed MS-specific antibody, fulfilment of this criterion remains uncertain.

#### 2.3.3 Direct correlation between autoantibody or T cell and disease activity

A demonstrated correlation between autoantibodies or T cell reactivity and disease is key to establishing autoimmune involvement. Significant correlations have been established between antibodies and disease severity in antisynthetase syndrome and hidradenitis suppurativa (89, 188).

Similar correlations have been suggested in MS (189). Indeed, it has been shown that the Igs titre in MS correlates with the degree of disability at the disease onset and development, with the extent of those correlations being dependent on other factors such as HLA haplotype (90, 91). In contrast, studies have shown no correlation between oligoclonal bands in clinically isolated syndromes and the risk of developing MS (92). Additionally, the results were not homogeneous in every patient, and the lack of well-identified autoantibodies specifically associated with MS makes this criterion difficult to validate. Further studies are crucial to confirm the prognostic values of these autoantibodies in MS.

This criterion also involves analysing the relationship between reactive T cells and disease activity, including relapses and new lesions. In SLE, for instance, the increase in circulating perforin or granzyme B-positive CD8^+^ T cells is correlated with disease activity (93).

Similarly, reactive T cells are more frequently found in people with MS experiencing flare-ups (94). While the frequency of autoreactive T cells may be elevated in people with MS relative to healthy controls, their presence in healthy individuals complicates the interpretation of MS as a classical AD (95). Various pro-inflammatory cytokines have been identified in the brain, CSF, and blood of people with MS, supporting the T cell activation (190, 191). Cytokines such as interferon-gamma (IFN-γ) and tumour necrosis factor-alpha (TNF-α) are notably elevated in people with MS and have been linked to exacerbated clinical symptoms and neurodegeneration (192, 193).

Lastly, the meaningful efficacy of immune-targeted therapies in MS, resulting in an average reduction of disease activity (e.g. relapse rate, disability accrual, MRI lesion) by blocking antibody secretion or T cell activation, supports these correlations (**Figure 1**) (122). At the same time, these treatments are not curative and act broadly on support function, often extending beyond antibody secretion or T-cell activation, underscoring challenges in specificity and in targeting disease-initiating processes.

Altogether, these results support a central role for the immune system in driving MS disease activity and worsening of disability. Nevertheless, caution is required to avoid overinterpretation that immune activation is the sole trigger of disease onset, as additional mechanisms may contribute to MS initiation and development.

#### 2.3.4 *In vitro* T cell proliferation and cytotoxicity in response to autoantigen or the target organ cells

Another way to model autoimmune mechanisms is by investigating *in vitro* T cell proliferation and cytotoxicity in response to autoantigens or target cells. This approach has been explored in the context of autoimmune disorders, such as T1D (96), and Guillain-Barré syndrome (99).

It is well established that reactive T cells are present in MS active lesions (194). There are also reports of cultured peripheral blood T cells from individuals with MS that can recognise myelin and oligodendrocyte peptides (72). However, the majority of studies investigating such mechanisms rely on the use of anti-myelin antibodies to elicit T cell responses, an approach that lacks disease specificity, as these targets are not unique to MS, and similar responses were observed in healthy controls (97, 98). Moreover, recent findings suggest that only citrullinated forms of MBP, but not native MBP, can induce T cell activation *in vitro* (195). This contrasts with other studies reporting minimal T cell reactivity to citrullinated MBP (196), highlighting inconsistencies in the literature. Despite the suggestion of new potential antigens (197), further work is required to validate these antigens as MS-specific.

Lastly, a preliminary investigation showed a greater T cell stimulation in response to brain homogenates from people with MS than from controls (100). However, although auspicious, this study is preliminary and requires a larger sample size and independent validation. Additionally, it has been shown that brain homogenates suppress lymphocyte proliferation *in vitro*, supporting its immune-privileged feature (101).

Although it is frequently observed that T cells play a key role in MS, the initial factors that trigger their activation remain unknown. Solving this mystery would confirm their involvement and thus provide a better understanding of their role in the initial MS onset.

#### 2.3.5 *In vitro* T cell transference to immunodeficient mice

Transferring disease-associated autoreactive T cells into naïve animals is a valuable tool to study how these cells initiate and sustain autoimmune attacks on the targeted organ, thereby resulting in pathologies. This approach has been explored in multiple ADs, e.g. peripheral blood mononuclear cells (PBMCs) transfer from individuals with systemic sclerosis induced tissue inflammation in mice (102), suggesting a role for immune cells in the pathogenesis.

In MS, transferring autoreactive T cells from EAE into naïve recipient animals succeeded in reproducing the disease (103). Although these experiments highlight that the transferability/induction of the disease involves autoreactive T cells, they remain specific to the antigen used for EAE induction. So far, most of the transferred T cells are against myelin proteins, which is unpredictable, strain-specific, and EAE does not reflect the full complexity of MS (20, 55).

Additionally, the transfer of human PBMCs from people with MS into humanised mice with disrupted BBB developed a unique model resembling MS immunopathology (104). In this model, CD4^+^ and CD8^+^ cells from both healthy and MS donors spontaneously invade the brain and spinal cord. However, spinal lesions and glial activation were only observed in the MS-transferred mice after immunisation with myelin peptides. Notably, the absence of observed demyelination, a hallmark of MS, raised questions about the necessity of myelin degradation and subsequent antigen generation, before immune-driven demyelination vis-à-vis a role in disease resolution.

The approaches discussed are fundamental criteria for establishing autoimmunity, and many efforts are bringing us closer to a definitive answer. Validating these direct proofs with confidence and robustness would be a significant step toward conclusively confirming the autoimmune basis of MS initiation.

## 3 Concluding remarks

This review critically re-examined the classification of MS as an AD by contrasting MS with conditions in which pathogenic autoimmunity is clearly established. Unlike previous reviews that have largely approached this question in conceptual or narrative terms (107, 110–112), we combined a working definition of AD with a criterion-by-criterion evaluation of MS, together with a direct comparison with well-established ADs. While immune dysregulation is undeniably a key component of MS, particularly in relapsing disease, the ongoing difficulty in identifying MS-specific autoantigens, coupled with increasing evidence of early neurodegeneration, raises questions about whether a purely autoimmune framework fully captures disease complexity. MS may therefore represent a complex interplay between neurodegeneration, metabolic disturbances, and immune activation. In this context, immune responses could represent both drivers and amplifiers of pathology, rather than the sole initiating mechanism.

By placing MS within the broader landscape of ADs, this review brings into focus conceptual gaps that are often acknowledged implicitly yet remain incompletely resolved. In an era marked by the reclassification of antibody-mediated demyelinating diseases (e.g. MOGAD, NMOSD), improved disease stratification, and the plateauing effectiveness of immune-targeted therapies in progressive MS, such reassessment is both timely and necessary. Importantly, this approach does not seek to diminish the role of the immune mechanisms in MS, but rather to systematically evaluate whether MS fulfils established criteria for AD. The frequent observation of shared genetic risk factors, comorbidities, and immune phenomena (e.g. environmental and viral factors) across MS and other ADs highlights overlapping immune processes, while simultaneously raising questions about disease specificity, a defining feature of classical autoimmunity. Studying MS alongside other ADs through a holistic, cross-disease framework may therefore help distinguish shared immune mechanisms from disease-specific pathogenic processes, with the potential to refine disease classification, sharpen biomarker discovery, and guide more targeted experimental and therapeutic strategies.

Rigorously testing the autoimmune criteria in MS is crucial for enhancing our comprehension of MS onset and pathogenesis. If cytodegeneration is indeed the primary trigger of MS, future studies should prioritise exploring the fundamental mechanisms that initiate this process and subsequent neurodegeneration. Ultimately, the ongoing debate about whether immune dysregulation is a cause or an effect of MS, the essential “chicken-and-egg” dilemma, remains unsettled. However, autoimmune inflammation likely represents only one segment of a broader and multifaceted pathophysiological process. Over the coming decade, MS research will benefit from efforts that integrate perspectives from neurodegeneration, metabolism, and immunology. By pursuing such a comprehensive approach, the field may move closer to clarifying the underlying mechanisms of MS onset and, ultimately, to developing interventions capable of altering the course of the disease.

## Glossary

AchR: Acetylcholine receptor
AD: Autoimmune disease
ADEM: Acute disseminated encephalomyelitis
AQP4: Aquaporin protein 4
BBB: Blood-brain barrier
Breg: Regulatory B cell
CD4+/CD8+: Cluster of differentiation 4/8
CNS: Central nervous system
CSF: Cerebrospinal fluid
DMT: Disease-modifying therapy
EAE: Experimental autoimmune encephalomyelitis
EBV: Epstein-Barr virus
GliaCAM: Glial cell adhesion molecule
GWAS: Genome-wide association studies
HLA: Human leukocyte antigen
HPV-6: Human papillomavirus 6
IFN-γ: Interferon-gamma
Igs: Immunoglobulins
JME: Japanese macaque encephalomyelitis
MBP: Myelin basic protein
MG: Myasthenia gravis
MHC II: Major histocompatibility complex 2
MOG: Myelin oligodendrocyte glycoprotein
MOGAD: Myelin oligodendrocyte glycoprotein-associated disease
MRI: Magnetic resonance imaging
MS: Multiple sclerosis
NMOSD: Neuromyelitis optica spectrum disease
NOD: Non-obese diabetic
PBMC: Peripheral blood mononuclear cell
PLP: Proteolipid protein
PPMS: Primary progressive multiple sclerosis
RA: Rheumatoid arthritis
RRMS: Relapsing-remitting multiple sclerosis
SLE: systemic lupus erythematosus
T1D: Type 1 diabetes
TCR: T cell receptor
TNF-α: Tumour necrosis factor-alpha
Treg: Regulatory T cell
